# Cognitive Triad and Depressive Symptoms in Adolescence: Specificity and Overlap

**DOI:** 10.1007/s10578-022-01323-w

**Published:** 2022-02-19

**Authors:** Igor Marchetti, Patrick Pössel

**Affiliations:** 1 Psychology Unit, Department of Life Sciences, University of Trieste, Via Edoardo Weiss, 21, 34128 Trieste, Italy; 2 Department of Counseling and Human Development, University of Louisville, Louisville, KY USA

**Keywords:** Cognitive triad, Depression, Commonality analysis, Meta-analysis

## Abstract

Adolescence is a critical period for the development of depressive symptoms and the understanding of vulnerability factors that facilitate their onset is pivotal. In this study, we focused on Beck’s cognitive triad, namely its three-fold structure, comprising views of the self, world, and future. Despite its crucial role in the cognitive theory of depression, the relationship between the cognitive triad and depressive symptoms in adolescence is still unclear. In our study, we adopted a meta-analytic commonality analysis approach, in order to clarify whether the three components of the triad overlap in accounting for depressive symptoms, or they show distinct profiles of association. By relying on six independent samples of early adolescents (age range = 13–14: n = 174, 66% female, n = 347, 41% female), mid adolescents (age range = 15–17: n = 304, 61% female; n = 92, 34% female), and late adolescents (age range = 18–21: n = 217, 84% female, n = 101, 56% female), we showed that the views of the self, world, and future substantially overlap in accounting for depressive symptoms, although specific areas of distinctiveness could be detected. Moreover, the association between the cognitive triad and depressive symptoms appeared to be a function of both the developmental phase and gender. Furthermore, the cognitive triad emerged as specifically related to symptoms related to negative mood, absence of positive mood, and negative appraisal of the past. These findings advance our understanding of cognitive vulnerability for depressive symptoms in adolescence.

**Supplementary Information:**

The online version contains supplementary material available at 10.1007/s10578-022-01323-w.

Affecting about 350 million people worldwide, depression is responsible for a large proportion of the burden of disease [1] and represents a main source of total disability adjusted life years [2]. In addition, depression affects ever younger age groups [3]. Rates of individuals impacted by depressive symptoms and major depression increase from approximately 2% during childhood [4] to 22–27% during early and mid adolescence [5], and 50% during late adolescence [6]. At the same time, gender differences in the depression rates develop during adolescence. Approximately 20% of girls and 7% of boys experience depressive symptoms before the end of their adolescence [7, 8]. This scenario raises the question whether the strength of the associations between some vulnerability factors and symptoms of depression changes from early to late adolescence and whether they are different depending on gender.

According to Beck’s cognitive theory [9], a crucial mechanism that facilitates depressive symptoms is the cognitive triad. This construct refers to a three-fold structure, consisting of negative views of the self, world, and future. Negative views of the self involve the presumption of oneself as inadequate, unworthy, or unlovable, while negative views of the world portray the world and others as both unjust and limiting the individual’s chances to accomplish their aims. Finally, negative views of the future include the perception of the future as consisting of hardships and as current difficulties being indefinitely maintained. The cognitive triad has been shown to be associated with both depressive symptoms in childhood [10] and adolescence [10, 11]. More recently, a network analysis study showed that, as compared to the other cognitive risk factors, such as negative cognitive style and dysfunctional attitudes, the cognitive triad is a strong proximal vulnerability for depressive symptoms in adolescence [12].

Despite its pivotal role, the theoretical and empirical status of the cognitive triad is still unclear. On the one hand, many theorists claim that the three components of the triad are largely overlapping, and not three distinct entities [13]. More specifically, the cognitive triad would refer to the views of the self as a whole and two specific aspects of the self, namely its future and its environment [14]. On the other hand, Beck [9] maintains that, although the three components of the triad correlate substantially, they are in fact separable constructs useful for clinical work. Finally, some studies showed that the negative views of the self and the future were the components most strongly correlated with depressive symptoms in adolescence [10, 15], while other studies also stressed the role of the negative views of the world in children and adolescents [16, 17].

In our study, we aimed to disentangle the degree to which each of the three components are specifically related to depressive symptoms in adolescents or whether they substantially overlap as vulnerability factors. Moreover, adolescence is not a static life-span period, but a phase characterized by marked differences between stages of development [5, 6] and genders [7]. Hence, we considered whether the association between the components of the cognitive triad and depressive symptoms were different between early adolescents (13- to 14-year-olds), mid adolescents (15- to 17-year-olds), and late adolescents (18- to 21-year-olds), and between boys and girls.

Furthermore, provided the marked heterogeneity of depression [18], we investigated whether the components of the cognitive triad were equally associated with all depressive symptoms or related to a subset of them. From a theoretical standpoint [13], cognitive triad is expected to be strongly associated with symptoms of low self-esteem, hopelessness, and negative evaluation of people. Moreover, previous studies reported that cognitive vulnerabilities and cognitive biases are specifically related to negative mood (i.e., sadness) and absence of positive mood (i.e., anhedonia), negative appraisal of the self (i.e., self-aversion and worthlessness), and negative appraisal of the past and the future (i.e., feelings of failure and pessimism) [19, 20]. Hence, we hypothesized that the cognitive triad is substantially associated with negative mood, lack of positive mood, feelings of past failures, worthlessness, hopelessness, and negative evaluation of people.

In order to reach these goals, we relied on commonality analysis, a sophisticated statistical method, which allowed us to disentangle the association pattern between the cognitive triad components and depressive symptoms [21]. It is worth mentioning that this method has successfully been applied to explore the link between cognitive vulnerability and depressive symptoms in previous studies [19, 20]. Finally, in order to provide results that are reliable and not sample-specific, we calculated a fixed-effect meta-analytic commonality analysis across six data sets, for a total pool of about 1250 adolescents.

## Methods

### Participants

Two samples of early adolescents were included in this study. The first sample consisted of 174 individuals (age = 14.64 ± 0.26 years old, age range = 13–14, female = 66%; European-American = 78%, African-American = 11%, other = 11%, Midwestern US, [22]), while the second sample listed 347 individuals (age = 13.93 ± 0.25 years old, age range = 13–14, female = 41%; European-American = 35%, African-American = 45%, other = 20%, Midwestern US, [11]). Two samples of mid adolescents were also recruited, consisting of 304 individuals (age = 15.67 ± 0.62 years old, age range = 15–17, female = 62%; European-American = 71%, African-American = 16%, other = 13%, Midwestern US, [22]) and 92 individuals (age = 15.08 ± 0.31 years old, age range = 15–17, female = 34%; European-American = 39%, African-American = 43%, other = 18%, Midwestern US, [11]). Finally, we relied on two groups of late adolescents, consisting of 217 individuals (age = 20 ± 0.7 years old; range = 18–21; female = 84%, Germany, [23]) and 101 individuals (age = 19.59 ± 1.08 years old; range = 18–21; female = 56%; European-American = 63%, African-American = 19%, other = 18%, Midwestern US, [24]).

Early and mid adolescents were recruited as follows [11, 22]. Guardians of all 9th and 10th grade students were informed about the study by mail, inviting their offspring to participate. If guardians consented, students were asked for their written assent to participate as well. The only formal exclusion criterion was poor proficiency in English. No reward for participation was offered. Late adolescents were recruited from universities in Germany [23] and the US [24]. No formal exclusion criterion was applied and participation was either voluntary or rewarded with course credits. Across samples, questionnaires were administered in groups, under the supervision of a trained researcher.

### Measures

#### Questionnaires

The cognitive triad was measured with the English [25] and German [26] Cognitive Triad Inventory (CTI [25]) and the English Cognitive Triad Inventory for Children (CTI-C [27]). Both CTI and CTI-C list 36 items, capturing the three components of Beck’s triad, namely the view of self (“I am a failure”), world (“The world is a very hostile place”), and future (“There is no reason for me to be hopeful about my future”). While the CTI was measured on a 7-point Likert scale, from “totally agree” to “totally disagree”, the CTI-C items were shown on a 3-point Likert scale, namely “yes”, “maybe”, “no”. Higher scores indicated a more positive view of the component that is being measured.

Depressive symptoms were measured with the English [28] and German [29] Center for Epidemiological Studies – Depression Scale (CESD), which is a self-report questionnaire listing 20 items on a 4-point Likert scale, from “rarely or none of the time” to “most or all of the time”. Higher scores represented a higher frequency of depressive symptoms. All the measures across the samples showed moderate to excellent internal reliability (Cronbach’s α range: 0.68—0.92).

### Statistical Analysis

**Fig. 1:**
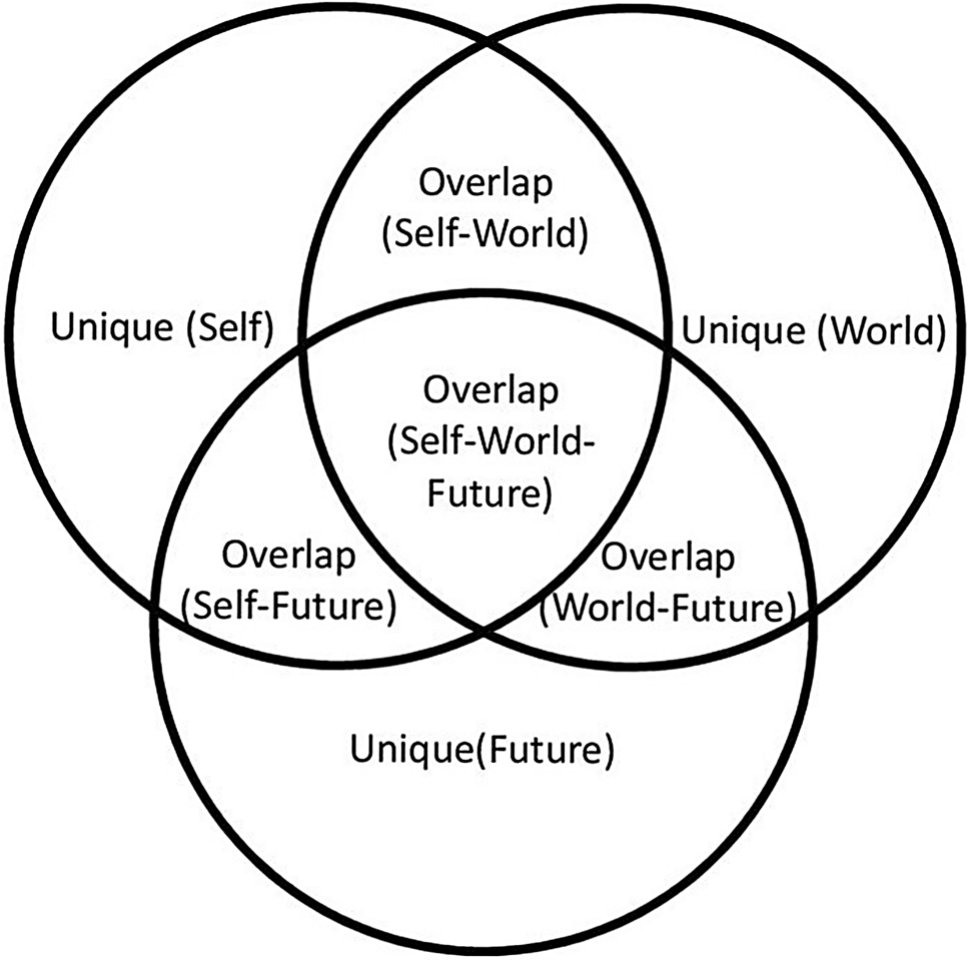
Commonality analysis with views of the self, world, and future as predictors and depressive symptoms as outcome

After reporting the descriptive statistics and Pearson’s correlations for each sample, we ran a series of commonality analysis (CA). CA is a variance partitioning technique aiming to decompose model fit (R^2^) into non-overlapping uniquely and commonly explained partitions [21]. When dealing with three predictors, commonality analysis yields seven partitions (Fig. 1), namely three partitions reflecting the amount of variance uniquely explained by the view of self (unique self), the view of world (unique world), or the view of future (unique future). Four partitions represent the overlap among the different components. For instance, the overlap among the view of the self, world, and future represents that amount of variance of depressive symptoms that they can explain interchangeably. It is worth stressing that all the partitions are quantified as the amount of explained variance and can be conveniently viewed as effect sizes (e.g., < 1% negligible, > 1% small, > 9% moderate, and > 25% large; [30]).

In line with guidelines calling for bootstrapping [31], we adopted percentile-based 95% two-tailed bootstrap confidence intervals (1000 bootstrap samples). At the level of each partition, we used bootstrap to quantify the precision of estimated parameter rather than to accomplish null-hypothesis significance testing.

Moreover, we integrated commonality analysis with meta-analysis and, by doing so, we could obtain a meta-analytic synthesis for each model across six samples. Assuming that there is one true population effect, which underlies all the studies included in the analysis, we performed a fixed-effect meta-analysis. The population effect was estimated using a weighted mean, where the weight assigned to each study was the inverse of that study’s variance. In each bootstrap sample, such fixed-effect could be estimated, and a 95% confidence interval for the common effect was obtained. Furthermore, when comparing the same partitions across two different models (i.e., unique component of the view of self for males vs. for females), we calculated the difference in unique and common meta-analytic effects between outcomes in every bootstrap sample, and similarly obtained a 95% confidence interval for the difference.

Current guidelines encourage adopting a meta-analytical approach whenever possible, in order to build a cumulative discipline [31]. It is worth remembering that meta-analysis is not a procedure that can be applied only after performing a large-scale systematic review of the literature, but also a helpful way to combine small-scale data sets and provide more trustworthy and reliable results [31]. In line with specific statistical recommendations, we meta-analytically combined different data sets, which were at our disposal. A similar approach has already been adopted in the field of depression research [19].

In conclusion, we followed a four-step analytic plan: (i) meta-analytic commonality analysis on depressive symptom total score; (ii) meta-analytic comparison between early, mid, and late adolescents; (iii) meta-analytic comparison between genders; (iv) meta-analytic commonality on each CESD depressive symptom.

## Results

### Descriptive and Correlation Analysis

Means, standard deviations, and Pearson’s correlations among the view of the self, world, and future, and depressive symptoms total score across six studies are reported in Table 1.

**Table 1:** Descriptive statistics and Pearson’s correlations across early, mid, and late adolescents

Variable	Early adolescents (EA) #1 (n = 174) M (SD)	Early adolescents (EA) #2 (n = 347) M (SD)	CTIC-S	CTIC-W	CTIC-F	CESD
CTIC-S	24.90 (4.87)	25.42 (4.31)		0.68	0.70	− 0.69
CTIC-W	23.72 (4.00)	23.49 (3.51)	0.79		0.61	− 0.61
CTIC-F	24.90 (4.97)	25.45 (3.99)	0.85	0.75		− 0.47
CESD	18.04 (11.95)	14.80 (10.85)	− 0.67	− 0.69	− 0.63	

Variable	Mid adolescents (MA) #1 (n = 304) M (SD)	Mid adolescents (MA) #2 (n = 92) M (SD)	CTIC-S	CTIC-W	CTIC-F	CESD
CTIC-S	23.65 (5.09)	25.37 (4.22)		0.69	0.75	− 0.70
CTIC-W	22.54 (4.02)	23.46 (3.46)	0.80		0.63	− 0.52
CTIC-F	23.52 (5.41)	25.39 (3.98)	0.87	0.78		− 0.58
CESD	19.57 (10.85)	20.14 (9.43)	− 0.66	− 0.68	− 0.57	

Variable	Late adolescents (LA) #1 (n = 217) M (SD)	Late adolescents (LA) #2 (n = 101) M (SD)	CTI-S	CTI-W	CTI-F	CESD
CTI-S	55.07 (8.62)	54.18 (9.32)		0.67	0.73	− 0.72
CTI-W	52.52 (6.81)	50.50 (7.11)	0.60		0.53	− 0.63
CTI-F	53.22 (6.46)	59.64 (9.13)	0.55	0.48		− 0.64
CESD	15.91 (9.97)	17.06 (12.05)	− 0.47	− 0.54	− 0.54	

Pearson’s correlations for studies #1 are shown below the diagonal. Pearson’s correlations for studies #2 are shown above the diagonal

*CTI(C)-S* view of the self, *CTI(C)-W* view of the world, *CTI(C)-F* view of the future, *CESD* depressive symptoms

### Meta-analytic Commonality Analysis on Depressive Symptoms Total Score

**Fig. 2:**
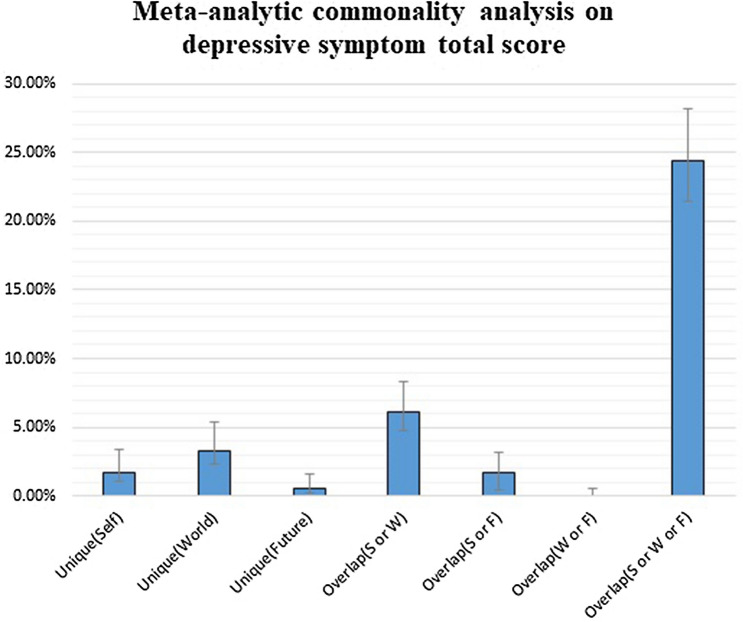
Meta-analytic commonality analysis across six independent samples, with views of the self, world, and future as predictors and depressive symptoms as outcome

The fixed-effect meta-analytic commonality analysis was performed across the six data sets, where the three components of the cognitive triad served as predictors and the depressive symptoms total score served as criterion (Table 2; Fig. 2). The analysis revealed that the largest component was the overlap among the view of the self, world, and future (24.38%, moderate-to-large effect). Other two components could account for a significant, but small amount of variance, namely the overlap between the view of self and world (6.13%, small effect) and the unique component of the view of world (3.24%, small effect). All the other components were negligible.

**Table 2:** Meta-analytic community analysis across six samples

Partition	Explained variance [95% confidence intervals]
Unique (Self)	1.75 [1.09; 3.35]
Unique (World)	3.24 [2.38; 5.36]
Unique (Future)	0.57 [0.26; 1.57]
Overlap (S or W)	6.13 [4.74; 8.34]
Overlap (S or F)	1.67 [0.48; 3.14]
Overlap (W or F)	− 0.01 [− 0.42; 0.57]
Overlap (S or W or F)	24.38 [21.44; 28.17]

*S* view of the self, *W* view of the world, *F* view of the future

### Meta-analytic Comparison Between Early, Mid, and Late Adolescence

**Fig. 3:**
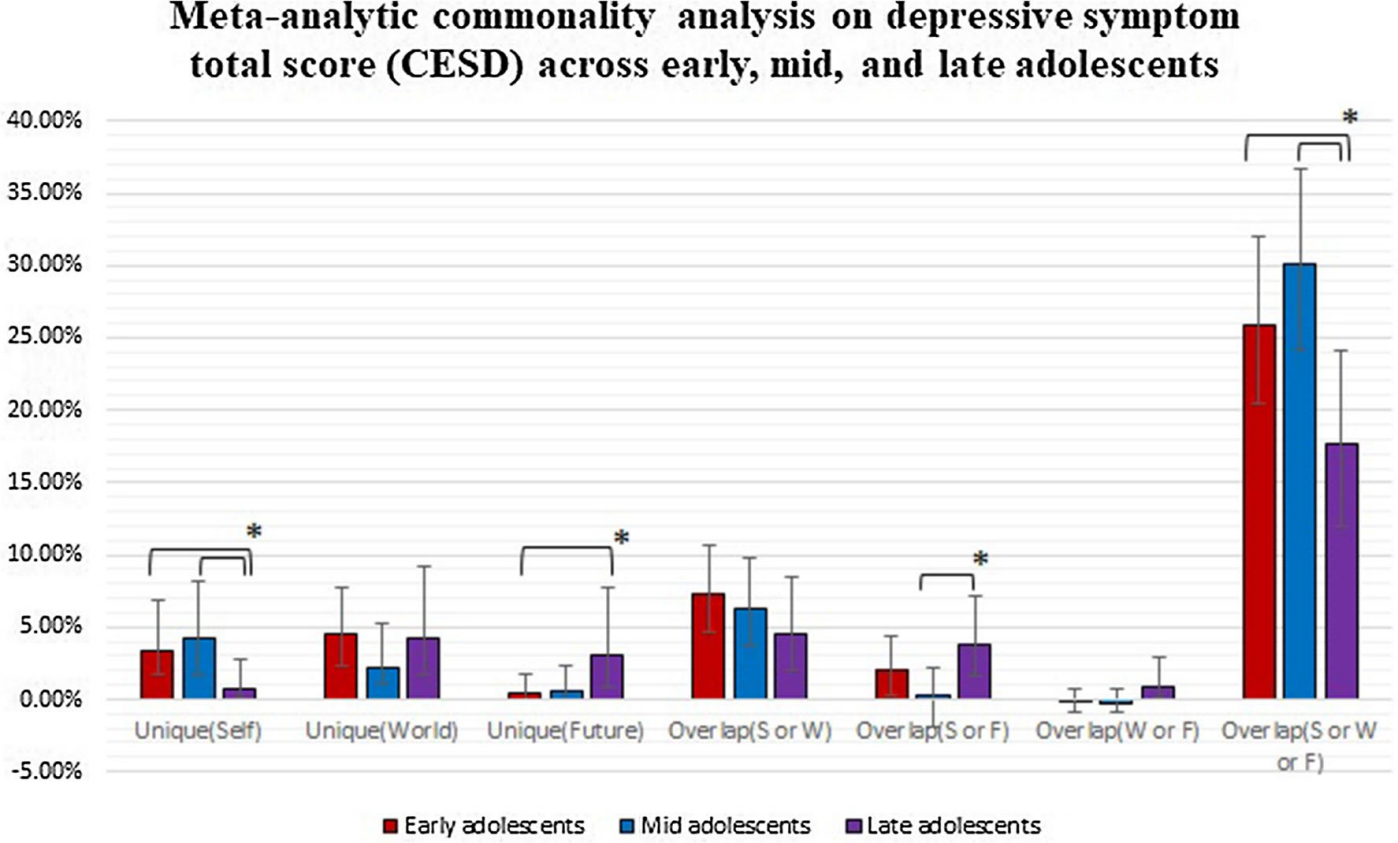
Meta-analytic commonality analysis across early, mid, and late adolescents

We compared the estimates of the commonality analysis between the groups of early, mid, and late adolescents (Fig. 3, Table S1). The overlap among the view of the self, world, and future was substantially larger in early and mid adolescents than in late adolescents (25.84% vs. 30.04% vs. 17.62%, respectively). Three other components were statistically different, although of negligible-to-small magnitude. The unique component of the view of self was larger in early and mid adolescents than in late adolescents (3.43% vs. 4.29% vs. 0.73%), while the unique component of the view of future was smaller in early adolescents than in late adolescents (0.39% vs. 3.07%). Finally, the overlap between the view of the self and the future was smaller in mid adolescents than in late adolescents (0.26% vs. 3.83%).

### Meta-analytic Comparison Between Male and Female Adolescents

We compared the commonality partitions between male and female adolescents (Table S2; Fig. S1). The unique component of the view of self was larger in males than in females (4.83% vs. 1.07%), while the unique component of the view of world was larger in females than in males (5.52% vs. 1.92%). Moreover, the overlap between the view of the self and the future was larger in females than in males (6.48% vs. 1.04%). The overlap among the view of the self, world, and future was substantially larger in females than in males (29.86% vs. 15.34%).

### Meta-analytic Commonality Analysis on Each CESD Depressive Symptom

Finally, we performed a meta-analytic commonality analysis on each of the twenty depressive symptoms, as listed in the CESD (Table S3). The overlap among the view of the self, world, and future was consistently the largest component, across all the symptoms. In detail, the overlap among the three components of the cognitive triad explained a moderate-to-large amount of variance of four symptoms, namely *feelings of failure* (18.81%), (*lack of*)* joy of life* (16.93%), (*lack of*)* happiness* (16.92%), *feeling depressed* (10.49%), and *cry spells* (9.14%).

## Discussion

In our study, we investigated whether the negative views of the self, world, and future overlapped in relation to depressive symptoms in adolescents, or they acted as distinct components of the cognitive triad. The meta-analytic synthesis of our results revealed that the way adolescents perceive their self, future, and environment substantially converge in relation to depressive symptoms, although areas of distinctiveness were detected.

The overlap among the views of the self, world, and future emerged as the largest partition in explaining depressive symptoms (~ 25%) and, more specifically, this partition was significantly larger in early and mid adolescents than in late adolescents (~ 26% vs. ~ 30% vs. ~ 18%). Interestingly, this finding could parallel a well-known developmental trajectory in adolescence, namely the increase of self-complexity and differentiation [32]. Previous studies have showed that during the later stages of adolescence the different components of the self (e.g., the way adolescents view themselves in the present, in relation to others, and the way they would like to be in the future) are less interconnected and dependent on one another [33]. Similarly, developmental psychopathology [34, 35] and empirical studies on the development of the brain [36] show that cognitive vulnerabilities emerge and become more differentiated over time, from early to late adolescence. In keeping with this, reduced differentiation among the components of the self is typically correlated with internalizing symptoms in adolescence [32].

Furthermore, female and male adolescents showed a substantially different profile. On the one hand, female adolescents reported a larger overlap of the three components of cognitive triad with depressive symptoms than male adolescents (~ 30% vs. ~ 15%), followed by larger overlap between the self and the world components (~ 6% vs. ~ 1%) and a larger unique component of the world (~ 6% vs. ~ 2%). On the other hand, male adolescent showed a larger unique component of the self, as compared to female adolescents (~ 5% vs. ~ 1%). Taken together, these findings suggest that the preeminent role of the social environment could be a key factor in understanding gender differences in the cognitive risk for depressive symptoms in adolescence. Previous work solidly demonstrated that adolescent girls are more sensitive than boys to social cues, due to gender-specific developmental differences (i.e., pubertal timing and body image [37]). Consistent with that, the sense of self-worth in boys is less dependent upon inputs from relational context than girls [38]. Hence, the way an adolescent navigates the social world may represent an important focus for understanding the functioning of depressive symptoms in this developmental phase and maybe a promising clinical target.

Furthermore, the influence of the view of the future was a function of age. In detail, the unique component of the future and the overlap between the view of the self and the future played a more important role in late adolescents, as compared to early and mid adolescents. This is in line with previous evidence showing that future orientation is increasingly prominent in adolescents aged 16–17 or older [39] and that adolescents who consider their personal future as gloomy and dismal are at high risk to develop depressive symptoms [40]. Although the analysis revealed that the magnitude of the effect is small, our meta-analytical synthesis provides initial support to interventions that aim to modify future orientation (i.e., [41]). Future studies should further explore the specific role of negative future in facilitating adolescent depressive symptoms.

It is worth stressing that the overlap among the three components of the cognitive triad was not equally associated with all the measured depressive symptoms. In particular, this partition was specifically linked to a negative appraisal of the past, negative mood, and absence of positive mood. On the one hand, this pattern of findings was substantially in line with our hypotheses and previous work on cognitive risk factors for depressive symptoms [19, 20]. On the other hand, the different components of the cognitive triad were not strongly associated with the depressive symptoms they were purportedly intended to measure, such as worthlessness and hopelessness. A possible explanation is that many of the symptoms measured with the CESD were phrased in a positive fashion (i.e., “I felt hopeful about the future”) instead of a negative fashion (i.e., “I feel my future is hopeless”), while the CTI scores consists of both positive and negative items [26]. Considering that psychometric research solidly showed that reverse items rarely function as the opposite of straightforward items [42], positive item phrasing may have blunted the degree of association between cognitive triad and specific depressive symptoms. Future studies should further investigate this topic, by including depressive symptom items phrased in both the negative and positive fashion.

Our study has limitations. First, only self-report measures were used and no other assessment method was adopted. While depressive symptoms could be evaluated by means of clinical interviews, no other methods are currently available to assess the three components of the cognitive triad [43]. Second, the age range was limited only to adolescence. While the cognitive triad is known to be already present in childhood [44], future studies should broaden the focus by also including early and late children. Third, all six included studies were of cross-sectional nature, hence no directionality could be derived. Future studies could evaluate the degree of specificity and overlap of the cognitive triad in accounting for future depressive symptoms. Fourth, the current study is a small-scale meta-analytical combination of studies that were at our disposal, while future research should focus on performing a large-scale systematic meta-analysis of evidence about the relationship between the cognitive triad and adolescent depressive symptoms.

To our knowledge, this is the first study that clarifies in a large sample of early, mid, and late adolescents the relationship between the cognitive triad and depressive symptoms, showing that a general overlap among the views of the self, world, and future as well as areas of distinctiveness are active. Moreover, both the developmental phase and gender seem to play an important role. Finally, our study revealed that the cognitive triad is specifically associated with experiencing negative mood and lack of positive mood as well as negatively appraising the past.

## Summary

The main goal of this study was to investigate the structure of cognitive vulnerability for depressive symptoms in adolescence. By focusing on Beck’s cognitive triad, we aimed to disentangle the specific pattern of association between depressive symptoms and negative view of the self, the world, and the future. To do so, we relied on commonality analysis, a sophisticated analytical approach, which allowed us to quantify the amount of specificity and commonality that the three components of the Beck’s cognitive triad shared with depressive symptoms. Moreover, in order to provide reliable and trustworthy results, we adopted a meta-analytical approach (i.e., meta-analytical commonality analysis), across six data sets of early, mid, and late adolescents (i.e., final sample of approximately 1250 individuals). The analysis revealed that the negative views of the self, the world, and the future substantially overlapped with depressive symptoms (i.e., approximately 25%). This effect was further qualified by both gender and age group. Overlap was indeed larger in early and mid adolescents as compared with late adolescents, with this result potentially reflecting the greater ability of late adolescents to differentiate emotional information related to the self. Moreover, girls showed greater overlap and greater unique contribution of the world component than boys did. Different socialization processes might explain these effects. Late adolescents showed a more prominent role of the view of the future, as compared to early and mid adolescents. Finally, the overlap among the three components of the Beck’s cognitive triad was markedly related to negative mood, lack of positive mood, and negative appraisal of the past.

## Supplementary Information

Below is the link to the electronic supplementary material.

Supplementary file1 (DOCX 123 kb)
