# Supplementary material for: Cognitive Triad and Depressive Symptoms in Adolescence: Specificity and Overlap
Source: Child Psychiatry Hum Dev. 2022 Feb 19;54(4):1209–17. doi: 10.1007/s10578-022-01323-w (PMC10272270; doi:10.1007/s10578-022-01323-w)

**Supplementary Material**

| *Table S1. Meta-analytic comparison across early, mid, and late adolescents. Explained variance, 95% bootstrap confidence intervals, and estimate difference.* | | | | | | |
| --- | --- | --- | --- | --- | --- | --- |
| Partition | Early Adolescents (n=521) | Mid Adolescents (n=396) | Late Adolescents (n=318) | Estimated difference EA-MA | Estimated difference EA-LA | Estimated difference MA-LA |
| Unique (Self) | 3.43% [1.82%; 6.88%] | 4.29% [1.70%; 8.23%] | 0.73% [0.13%; 3.79%] | -0.85% [-5.19%; 3.67] | **2.70% [0.01%; 6.02%]** | **3.56% [0.22%; 7.43%]** |
| Unique (World) | 4.53% [2.31%; 7.78%] | 2.13% [1.15%; 5.29%] | 4.30% [1.81%; 9.18%] | 2.40% [-1.48%; 5.96%] | 0.23% [-4.87%; 4.08%] | -2.17% [-6.80%; 1.44%] |
| Unique (Future) | 0.39% [0.04%; 1.76%] | 0.59% [0.05%; 2.33%] | 3.07% [0.83%; 7.81%] | -0.20% [-1.92%; 1.15%] | **-2.68% [-7.13%; -0.01%]** | -2.48% [-7.04%; 0.38%] |
| Overlap (S or W) | 7.35% [4.66%; 10.73%] | 6.24% [3.70%; 9.83%] | 4.57% [2.01%; 8.45%] | 1.11% [-3.75%; 5.37%] | 2.78% [-2.30%; 7.18%] | 1.67% [-2.82%; 6.36%] |
| Overlap (S or F) | 2.02% [0.30%; 4.38%] | 0.26% [-1.89%; 2.21%] | 3.83% [1.54%; 7.18%] | 1.76% [-0.97%; 4.90%] | -1.82% [-5.50%; 1.48%] | **-3.57% [-7.39%; -0.38%]** |
| Overlap (W or F) | -0.05% [-0.84%; 0.66%] | -0.26% [-0.95%; 0.71%] | 0.85% [0.11%; 2.95%] | 0.21% [-1.08%; 1.16%] | -0.90% [-3.18%; 0.10%] | -1.11% [-3.23%; 0.08%] |
| Overlap (S or W or F) | 25.84% [20.49%; 31.39%] | 30.04% [24.06%; 36.64%] | 17.62% [11.97%; 24.14%] | -4.20% [-12.70%; 4.51%] | **8.22% [0.37%; 16.31%]** | **12.42% [4.27%; 20.35%]** |
| *Note:* S: view of the self; W: view of the world; F: view of the future. Bold estimated differences are statistically significant (*p* < 0.05). | | | | | | |

| *Table S2. Meta-analytic comparison between male and female adolescents. Explained variance, 95% bootstrap confidence intervals, and estimate difference.* | | | |
| --- | --- | --- | --- |
| Partition | Male Adolescents (n=519) | Female Adolescents (n=715) | Estimated difference Male-Female |
| Unique (Self) | 4.83%[3.00%; 9.65%] | 1.07% [0.59%; 2.75%] | **3.75% [1.33%; 8.38%]** |
| Unique (World) | 1.92% [0.95%; 5.07%] | 5.52% [4.05%; 8.36%] | **-3.60% [-6.32%; -0.27%]** |
| Unique (Future) | 0.38% [0.12%; 1.88%] | 1.06% [0.53%; 2.68%] | -0.68% [-2.21%; 0.71%] |
| Overlap (S or W) | 1.04% [-0.62%; 2.89%] | 6.48% [4.47%; 9.52%] | **-5.44% [-8.80%; -2.78%]** |
| Overlap (S or F) | 3.46% [1.23%; 6.90%] | 0.83% [-0.44%; 2.45%] | 2.63% [-0.25%; 6.21%] |
| Overlap (W or F) | -0.03% [-0.64%; 0.79%] | 0.05% [-0.61%; 0.69%] | -0.08% [-0.93%; 1.00%] |
| Overlap (S or W or F) | 15.34% [11.38%; 20.04%] | 29.86% [25.76%; 34.41%] | **-14.52% [-20.39%; -8.08%]** |
| *Note:* S: view of the self; W: view of the world; F: view of the future. Bold estimated differences are statistically significant (*p* < 0.05). | | | |

| *Table S3. Meta-analytic community analysis across the six samples on CESD symptoms.* | | | | | | | | |
| --- | --- | --- | --- | --- | --- | --- | --- | --- |
|  | Unique (Self) | Unique (World) | Unique (Future) | Overlap (S or W) | Overlap (S or F) | Overlap (W or F) | Overlap (S or W or F) |  |
| Bothered (CESD1) | 0.82% | 1.27% | 0.33% | 1.29% | 0.29% | 0.13% | 4.63% |  |
| Eat (CESD2) | 0.39% | 1.33% | 0.35% | 0.99% | 0.22% | -0.03% | 1.83% |  |
| Blues (CESD3) | 0.81% | 1.55% | 1.00% | 1.73% | 0.75% | 0.08% | 6.50% |  |
| Good as other people (CESD4) | 2.50% | 0.66% | 0.54% | 0.76% | 4.22% | 0.06% | 8.49% |  |
| Mind on things (CESD5) | 0.62% | 0.90% | 0.33% | 1.52% | 0.70% | 0.09% | 6.47% |  |
| Depressed (CESD6) | 0.66% | 2.06% | 1.82% | 2.24% | -0.16% | -0.16% | 10.49% |  |
| Effort (CESD7) | 0.72% | 0.25% | 0.52% | -0.01% | 0.94% | 0.00% | 1.32% |  |
| Hopeful (CESD8) | 0.27% | 0.26% | 2.60% | 0.12% | 2.17% | 0.36% | 7.33% |  |
| Failure (CESD9) | 4.47% | 0.31% | 1.39% | 1.62% | 5.21% | 0.04% | 18.81% |  |
| Fearful (CESD10) | 1.08% | 1.36% | 1.24% | 2.60% | 1.17% | -0.23% | 2.43% |  |
| Sleep (CESD11) | 0.45% | 2.18% | 0.22% | 1.56% | 0.14% | 0.00% | 3.79% |  |
| Happy (CESD12) | 0.52% | 0.99% | 0.64% | 1.79% | 2.85% | 0.31% | 16.92% |  |
| Talked (CESD13) | 1.13% | 0.35% | 0.29% | 0.37% | 0.34% | -0.01% | 3.78% |  |
| Lonely (CESD14) | 0.73% | 2.86% | 1.15% | 3.98% | 0.01% | -0.24% | 7.29% |  |
| People unfriendly (CESD15) | 0.93% | 2.84% | 1.09% | 3.71% | -0.18% | -0.30% | 4.99% |  |
| Enjoy life (CESD16) | 0.88% | 0.78% | 0.53% | 1.18% | 2.60% | 0.51% | 16.93% |  |
| Cry (CESD17) | 1.71% | 0.26% | 0.34% | 1.54% | 1.55% | 0.03% | 9.14% |  |
| Sad (CESD18) | 0.74% | 2.63% | 1.46% | 2.74% | -0.11% | -0.34% | 3.89% |  |
| People dislike me (CESD19) | 1.83% | 3.89% | 1.33% | 6.01% | -0.68% | -0.55% | 6.09% |  |
| Get going (CESD20) | 2.05% | 1.03% | 1.51% | 2.07% | 0.14% | -0.37% | 5.22% |  |
| *Note:* S: view of the self; W: view of the world; F: view of the future. | | | | | | | |  |
|  |  |  |  |  |  |  |  |  |

**Figure S1.** Meta-analytic commonality analysis across males and females.


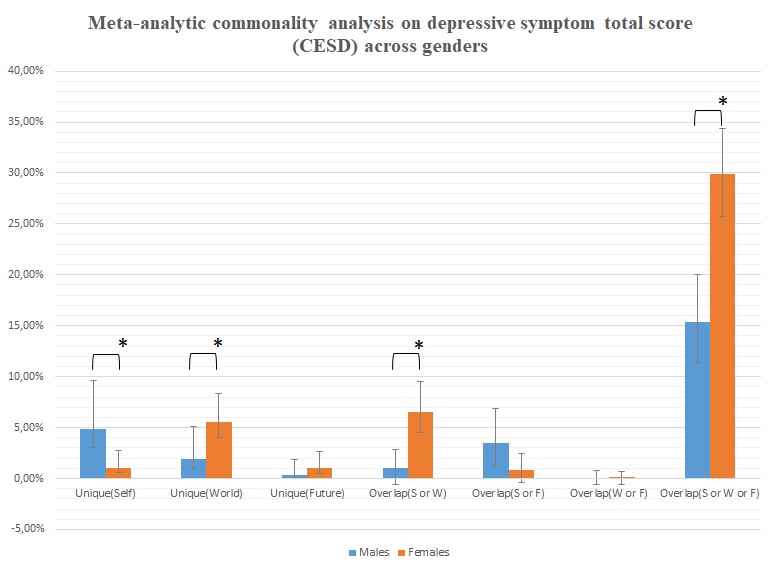

Supplement: Supplementary file 1 — Supplementary file1 (DOCX 123 kb) [file 10578_2022_1323_MOESM1_ESM.docx]
